# The perception of the stereo effect in bilateral and bimodal cochlear implant users and its contribution to music enjoyment

**DOI:** 10.1371/journal.pone.0235435

**Published:** 2020-07-06

**Authors:** Andreas Buechner, Benjamin Krueger, Silke Klawitter, Denise Zimmermann, Stefan Fredelake, Inga Holube

**Affiliations:** 1 Medical University of Hanover, Hanover, Germany; 2 Cluster of Excellence Hearing4all, Germany; 3 Advanced Bionics GmbH, European Research Center, Hanover, Germany; 4 Institute of Hearing Technology and Audiology, Jade University of Applied Sciences, Oldenburg, Germany; University of Miami School of Medicine, UNITED STATES

## Abstract

**Objectives:**

In this clinical study, stereo perception of music samples and its contribution to music enjoyment in CI users is investigated. It is studied in free field as well as direct audio presentation.

**Methods:**

20 bilateral and 9 bimodal CI users performed stereo detection tests and music enjoyment ratings. Music was presented either in mono or in stereo in free field or with direct audio presentation. Stereo detection was assessed with a 3-AFC paradigm. Music enjoyment was studied with scale ratings.

**Results:**

For bilateral CI users, stereo detection increased from 52% correct in free field to 86% with direct audio presentation. Increased music enjoyment with improved stereo detection was obtained. Bimodal CI users could not identify stereo sounds. Music enjoyment did not increase for stereo presentations in bimodal subjects.

**Discussion:**

For bilateral CI users, improved stereo detection might increase music enjoyment with direct audio presentation, which is likely due to bypassing the room acoustics. In bimodal CI users, no clear improvement was found, which is likely attributed due to the different hearing losses and therefore individually different interaural frequency overlaps between the hearing aid and the cochlear implant.

**Conclusion:**

Direct audio presentation is an efficient method to improve music enjoyment in bilateral CI users.

## Introduction

Cochlear implantation allows access to sounds in patients with severe to profound deafness. Many patients with a cochlear implant (CI) device have near perfect speech perception in quiet [1], although performance is significantly reduced in noise [2]. Technological evolution and the expansion of the CI inclusion criteria have resulted in improved speech perception performance [3]. With this improvement in sound perception, music enjoyment has become the next goal of many CI users. The quality of music presentation by the CI system and its perception by the CI user is still poor [4–6]. Whilst rhythm can be accurately detected by CI users with similar performance to normal hearing (NH) listeners [6–8], the perception of more complex structures such as melodies and timbre is severely reduced [6, 9, 10, 11]. In addition, CI users performed even worse than subjects with severe to profound hearing loss who used hearing aids (HA) although they were CI candidates. Indeed, the HA users had improved pitch discrimination and melody recognition than CI users indicating that the HA provided more reliable pitch information than CIs [11]. Though many CI users do report enjoying listening to music, the pleasure is reduced compared to NH listeners [12–16].

In NH listeners, stereo sound is the preferred format for listening to music as it provides features such as width, surrounding and spaciousness to a sound [17]. In order to achieve true stereo, two channels of information must be provided to the listener, which differ in level and phase. Those two channels are combined by the auditory system to produce a directional picture of the sound, a virtual sound source. The psychoacoustic mechanism underlying this phenomenon is the summing localization that occurs when two coherent sound waves that differ only in level and time, arrive at the ears. The interaural level differences (ILD) and interaural time differences (ITD) are processed by the auditory system so that only one sound source is perceived. Note, that stereophonic presentation must be distinguished from traditional binaural presentation. Stereophonic presentation uses two loudspeakers that are both heard in each ear. In contrast, traditional binaural presentation requires recordings at the eardrums of an auditor or dummy head and presenting these two recordings via headphones to the listener, i.e., the signal from the left ear is not heard on the right ear and vice versa. Though ILD and ITD perception are important mechanisms for both binaural and stereo perception, it is not the same in both listening modalities [18, 19]. While several traditional studies on binaural ILD and ITD perception have been conducted [e.g. 20–24], only little literature exists on CI users’ ability on stereo perception.

CI users with either a second implant or contralateral hearing aid (HA) have the possibility of accessing stereo sound and, therefore, enhance their music enjoyment. It was shown that the dichotic presentation of music pieces in this group, presenting bass to the one and treble to the other CI ear provided small significant improvements in subjective ratings based on clarity and preference [25]. This improvement was explained by an increase of the perceptual distance that occurred through lateralization streaming. However, ILD and ITD cues, the underlying cues enabling stereo sound perception, are disrupted by the sound processing strategies applied by the speech processor and their perception in CI users is poor [26, 27].

This clinical study evaluated whether stereo listening is possible in bilateral and bimodal CI users and whether it contributes to their music enjoyment. For this purpose, music was presented as stereo and mono variations via loudspeakers in free field as well as with direct audio input using the DirectConnect or the ComPilot of the Advanced Bionics system, respectively.

## Methods

### Study population

20 bilateral CI users (10 females and 10 males) between 41 and 80 years (average: 58.2 years) old were recruited. Furthermore, 9 bimodal CI users (4 females and 5 males) between 40 and 77 years (average: 62.7 years) old took part. They were recruited from the MHH clinical patient pool between 5th Nov 2014 and 27th Jun 2016. All had a postlingual onset of deafness and except for one were implanted with an Advanced Bionics HiRes90K or Clarion CII system, with at least 6 months of implant use. One had a C1 and HiRes90K device. Audiological and etiological information for the bilateral and bimodal group is shown in Tables 1 and 2. Furthermore, both tables show the occurrence of music listening per week, which was assessed by questionnaires that the patients filled prior experiments. For the bimodal users, the hearing loss is measured as the pure tone average of the audiometric thresholds in dB HL at the frequencies 500, 1000, 2000 and 4000 Hz of the HA ear. To ensure the same HA in all bimodal users, they were fitted with a Phonak Naída UP and the bimodal fitting formula at least 4 weeks prior to the experiment to ensure an acclimatization with the new HA in a take-home phase. No further modification were made to the prescribed amplification. The subjects were randomly divided into two groups: the DirectConnect (DC) group and the ComPilot (CP) group reflecting the different types of direct audio input (see Section Devices).

**Table 1:** Demographics of the bilateral CI users.

ID	Age	Duration of deafness (y)	Length of implant use 1^st^ ear (y)	Difference between length of implant use 1^st^ and 2^nd^ ear (y)	SI left (%)	SI right (%)	SI bilateral (%)	Music listening (x/week)	Group
01	60.9	25.9	7.9	2.0	90	95	100	<5	DC
02	50.7	19.7	6.2	4.2	85	60	75	5–10	DC
03	59.4	45.9	7.0	3.7	80	90	95	5–10	DC
04	49.3	32.7	11.2	3.4	90	85	95	10–20	DC
05	40.6	21.6	7.4	2.7	75	80	80	>20	DC
06	53.9	29.9	14.1	0.0	60	85	90	>20	DC
07	60.6	46.6	3.3	0.7	55	70	80	5–10	CP
08	60.1	23.7	7.2	1.3	60	10	45	<5	CP
09	63.9	33.1	1.9	1.5	60	65	75	5–10	CP
10	58.0	42.9	8.1	1.6	90	80	90	5–10	DC
11	57.9	12.5	5.8	0.9	35	35	50	5–10	CP
12	58.3	3.0	8.5	3.9	55	70	60	<5	CP
13	66.8	0.0	5.0	1.4	95	70	70	5–10	CP
14	79.7	0.1	7.5	0.0	50	50	40	<5	CP
15	51.3	0.8	7.0	3.1	55	50	55	>20	CP
16	51.9	23.7	13.8	2.6	80	15	70	5–10	CP
17	49.2	10.6	13.9	0.0	80	65	100	5–10	DC
18	51.6	4.9	14.8	6.1	50	55	70	<5	CP
19	72.7	6.5	8.0	5.1	90	85	95	<5	DC
20	66.9	7.1	21.7	18.5	70	75	85	NA	DC

SI: speech intelligibility score in % correct of Freiburg monosyllables measured with 65 dB presentation level, Music listening: occurrence of music listening per week in times per week, DC: DirectConnect group, CP: ComPilot group

**Table 2:** Demographics of the bimodal CI users.

ID	Age	Duration of deafness (y)	Length of implant use (y)	PTA (dB)	SI CI only (%)	SI bimodal (%)	Music listening (x/week)	Group
21	77.4	16.6	6.3	104	40	65	10–20	DC
22	70.0	0.0	2.5	88	70	80	5–10	CP
23	63.3	0.0	5.4	65	100	95	5–10	DC
24	65.1	6.4	6.7	90	75	95	5–10	CP
25	66.8	1.5	3.6	81	90	100	5–10	DC
26	53.0	9.2	2.3	78	80	95	5–10	CP
27	40.0	1.6	13.9	100	95	90	5–10	CP
28	69.3	0.0	5.6	75	80	95	5–10	DC
29	60.0	0.0	5.6	63	80	95	>20	CP

Pure tone average (PTA) is defined as the average of the hearing loss at the frequencies 500, 1000, 2000, and 4000 Hz, SI: speech intelligibility score in % correct of Freiburg monosyllables measured with 65 dB presentation level, Music listening: occurrence of music listening per week in times per week, DC: DirectConnect group, CP: ComPilot group

In addition, 10 NH listeners (5 females and 5 males) were recruited and served as a reference to the CI users. Ages ranged from 24 to 63 years (average: 40.7 years).

The study was approved by the local ethics commission of the Hanover Medical University, Germany. The subjects participating in the study signed a written consent form and a data release form.

### Devices

#### Harmony speech processor

The Harmony processor is an Advanced Bionics speech processor, worn behind the ear. The T-Mic is an ear hook with an integrated microphone that is located in front of the ear canal and picks up sounds. Sounds are further processed and provided to the implant beneath the skin. The T-Mic of the Harmony can be replaced by the DirectConnect, which is an another ear hook that can be wirely connected to auxiliary audio devices such as a sound card with a standard audio cable providing a direct audio input.

#### Naída CI speech processor

The Naída CI is a further development of the Harmony processor with more features such as advanced noise reduction, which are all not relevant for this clinical study. As the Naída CI does not offer a DirectConnect, the ComPilot was used to provide a wireless direct audio input.

#### Naída UP hearing aid

The Naída UP hearing aid was fitted with a bimodal fitting formula [28, 29]. The bimodal fitting formula consists of two major elements. First, the prescribed amplification was calculated using the model of effective audibility to ensure audibility of soft speech in the aidable range only. In addition, no gain was provided for frequencies within dead regions that were estimated from the audiogram, i.e., a reduced frequency bandwidth of the gain was applied if the slope of the hearing loss was more than 35 dB per octave and the high-frequency hearing loss exceeded 85 dB HL or if the hearing loss was more than 110 dB HL [29]. Second, the compression input-output function as well as the time constants were aligned to the AGC of the CI processor to ensure same loudness and dynamic characteristics between the HA and the CI [29]. Direct audio input to the HA was provided either with a 3 pin Euro plug, that was directly wired to a sound card or with the ComPilot for wireless transmission using a standard Bluetooth audio transmitter.

#### Wired audio input: DirectConnect

The DirectConnect (DC) is an ear hook for the Harmony speech processor and allows to connect the CI to auxiliary audio devices that have a standard audio output socket such as in sound cards. It applies the audio signal as voltage fluctuations from the socket directly to the auxiliary input of the Harmony processors and does not apply any audio coding. For the DC group, the left and right channel of sound card output was fed into the left and right processors using standard audio cables. In the bimodal case, one channel of the sound card output was fed into the HA using the 3 pin Euro plug. For the sake of simplicity, the bimodal case will also be entitled with DirectConnect or DC in the following.

#### Wireless audio transmission: ComPilot

The ComPilot (CP) is a wireless accessory for the Naída CI processor as well as the Naída UP HA and transmits audio signals to both devices in stereo sound. The CP makes use of audio coding that requires audio compression to account for Bluetooth bandwidth limitations and processing power. For the CP group, the left and right channels of the sound card output were fed into a standard A2DP Bluetooth audio transmitter that sent the signals wirelessly to the CP, which again transmitted the signals via the HiBan link to the Naída CI processors for bilateral CI users. In the bimodal case, the CP transmitted one channel to the Naída CI and one to the Naída UP HA.

### Test setup

#### Free field

All subjects were seated in a room that was sound damped and had low reverberation. Three loudspeakers were used with the left and right speakers and the chair for the subject creating an equilateral triangle with 1.2 m distance between the speakers and the subject. The third loudspeaker was placed the middle of the left and right speakers and was in a distance of approximately 1 m to the subject. All speakers were calibrated to the same sound pressure level at the position of the listener. For the experiments in free field the CI users were listening with their own clinical processors in their clinical program.

#### Direct audio presentation

For the direct audio presentation, the presentation modes differed for NH, bilateral and bimodal CI users.

The CI speech processor microphones were muted and only the signal provided by the DC or CP was heard. The same applied to the hearing aid in bimodal CI users, i.e., the microphone was muted and only the signal from the 3 pin Euro plug (DC) or CP was heard. Music was presented via a RME Babyface sound card. In all cases, the level was calibrated to the same sound pressure level as in the free field.

NH listeners were listening to the music via AKG K271 headphones that were connected to the same sound card and calibrated to the same sound pressure level as in the free field.

### Signals

Extracts from 25 different music pieces were used for the measurement. The music was commercially available music from different genres such as classic, pop, rock etc. in order to reflect a wide range of subjective music preference within controlled lab situations. Each music piece had a length between 5.5 and 11.4 s (median 9.4 s) and the long-term average level varied between 44.9 and 64.4 dB SPL (median 59.3 dB SPL). No level alignment was performed to have a realistic representation of music level distribution. All pieces were in stereo. Mono was obtained as the mean between the left and the right channel. The stereo signal was presented from the left and right speakers, the mono signal from center only and the double-mono signal with the mono signal from the left and right speakers. The motivation for this double-mono signal was to act as a control to make sure that the CI users truly perceived a stereo effect, when the stereo signal was presented in the free field and not only a summed sound from two separated loudspeakers presenting identical signals. For free field presentation, the sound pressure level of each music piece was roved within a corridor of ±3 dB to remove any possible bias from level-differences between stereo, mono and double-mono versions that occur due to the addition of the waveforms from both loudspeakers. For direct audio, the left and right channels of the stereo signal were presented to the left and right ears directly (dichotic presentation) and mono was presented to both ears simultaneously (diotic presentation). Note that there is no difference between mono and double-mono in the direct audio presentation. For one additional experiment with direct audio in bimodal users, the left and right channels were only streamed to the respective left and right ear to assess the music enjoyment from HA only, CI only and both devices together.

### Experiments

For bilateral CI users and NH listeners two music listening experiments were performed: first, stereo detection and second, music enjoyment rating. Bimodal CI users performed a third experiment on music enjoyment rating with HA alone, CI alone and both devices together. Stereo detection and music enjoyment were assessed in different tasks, because it was assumed that detection and enjoyment were different aspects of music perception. The presentation order of each condition as well as of each song was chosen randomly. Note that for each trial, the same music piece was presented in stereo, mono and double-mono. In all experiments, a touchscreen was used to collect the responses.

#### Experiment 1: Stereo detection

Stereo detection in free field was performed with the stereo, mono and double-mono signals. In contrast, with direct audio presentation the stereo signal was used once and the mono signal twice to have the same chance level as in free field.

The listener was instructed to listen to the different versions of each music piece that were presented in a 3-AFC paradigm in a random order and to indicate the stereo piece with a graphical user interface that had three push-buttons labelled as A, B and C. No repetitions after the presentation were possible.

#### Experiment 2 and 3 music enjoyment

Music enjoyment rating in free field was performed with the stereo, mono and double-mono signals. For Experiment 2 with direct audio, the signal was presented only once as a stereo signal and once as a mono signal to reduce measurement time. For Experiment 3, only the stereo signals with direct audio presentation were used and the left, right or both channels were presented to the respective ears.

The listener was instructed to listen to the different versions of the same music piece and to rate the music enjoyment for each version. For this purpose, a rating procedure with slider that was based on the MUSHRA scaling procedure was used [30]. Each slider represented one condition. The position of each slider was transformed linearly into a number between 0 and 100. The listeners had the option to repeat each presentation.

## Results

### Experiment 1: Stereo detection

**Fig 1:**
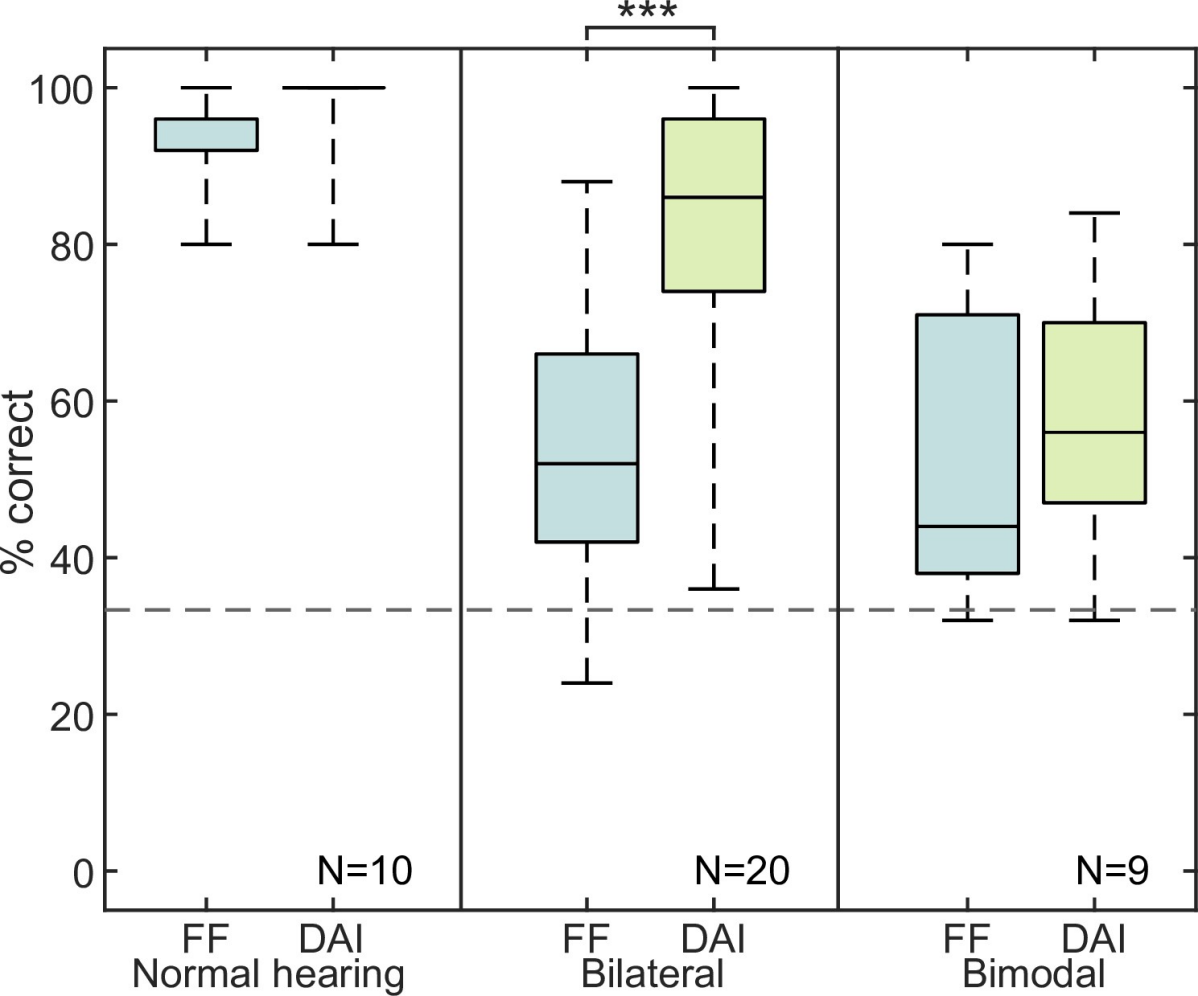
Results of experiment 1. Stereo detection in % correct for normal hearing listeners, bilateral and bimodal CI users for free field (FF) as well as direct audio input (DAI). Dashed line shows the chance level.

Fig 1 shows the number of correct responses plotted as boxplots for NH listeners (left), bilateral (middle) and bimodal (right) CI users tested in free field (FF) and direct audio input (DAI). Chance level is indicated with a dashed line.

In the NH group, the median value for correct identification of the stereo piece was 96% in free field. With headphone presentation, median performance reached 100%. The bilateral CI user group detected the stereo piece correctly in 52% of the time in the free field. Using the direct audio input, the correct detection rate increased up to 86%. Wilcoxon statistics with a Bonferroni-Holm correction revealed a significance effect of the presentation for the bilateral group (p<0.001). For the bimodal CI user group in the free field the median detection rate was 44%, which changed to 56% with direct audio presentation. The difference in detection rate in bimodal CI users was not significant.

**Fig 2:**
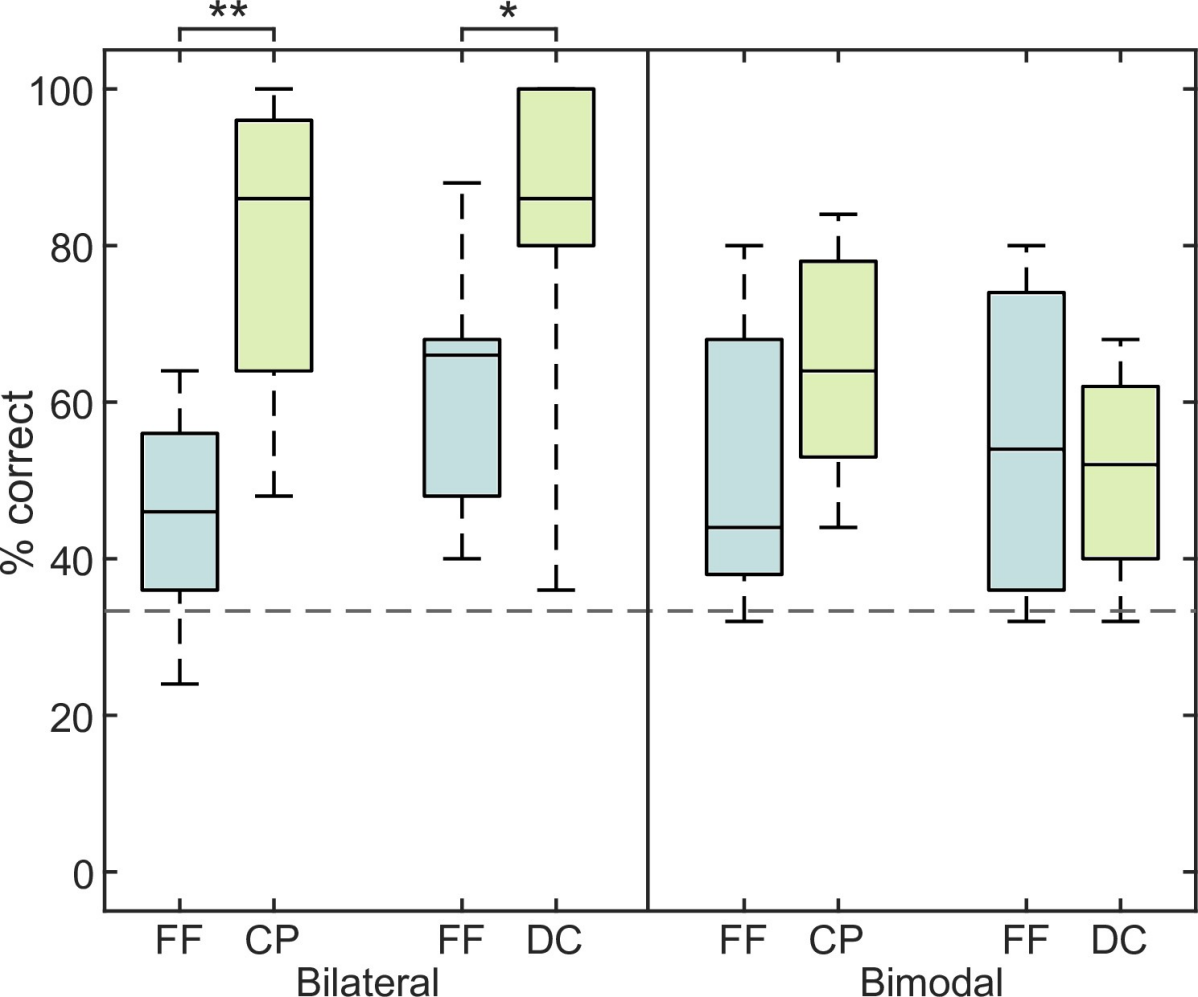
Results of experiment 1 with the groups separated. Stereo detection in % correct in free field (FF) and ComPilot (CP) and DirectConnect (DC) of the bilateral (left half) and bimodal (right half) CI users.

To assess any difference between the wireless CP and wired DC as audio input, the pooled CI user data from Fig 1 are plotted for both groups separately in Fig 2.

In the bilateral CP group, the stereo piece was detected 46% correctly in the free field. Using the CP, the correct detection rate increased up to 86%. For the bilateral DC group, the detection rate was 66% in free field and 86% with DC. Statistical significances between free field and direct audio presentation were tested for both bilateral groups with Wilcoxon statistics with a Bonferroni-Holm correction and revealed p-values of p < 0.01 for the CP group and p < 0.05 for the DC group. The bimodal CP group detected 44% correct in free field and 64% with the CP. The bimodal DC group detected 54% in free field and 52% with the DC. No statistical differences in stereo detection rate were found for the bimodal group.

### Experiment 2 and 3: Music enjoyment

To assess music enjoyment, median scale values for the 25 music pieces rated by each subject, were calculated for each condition.

**Fig 3:**
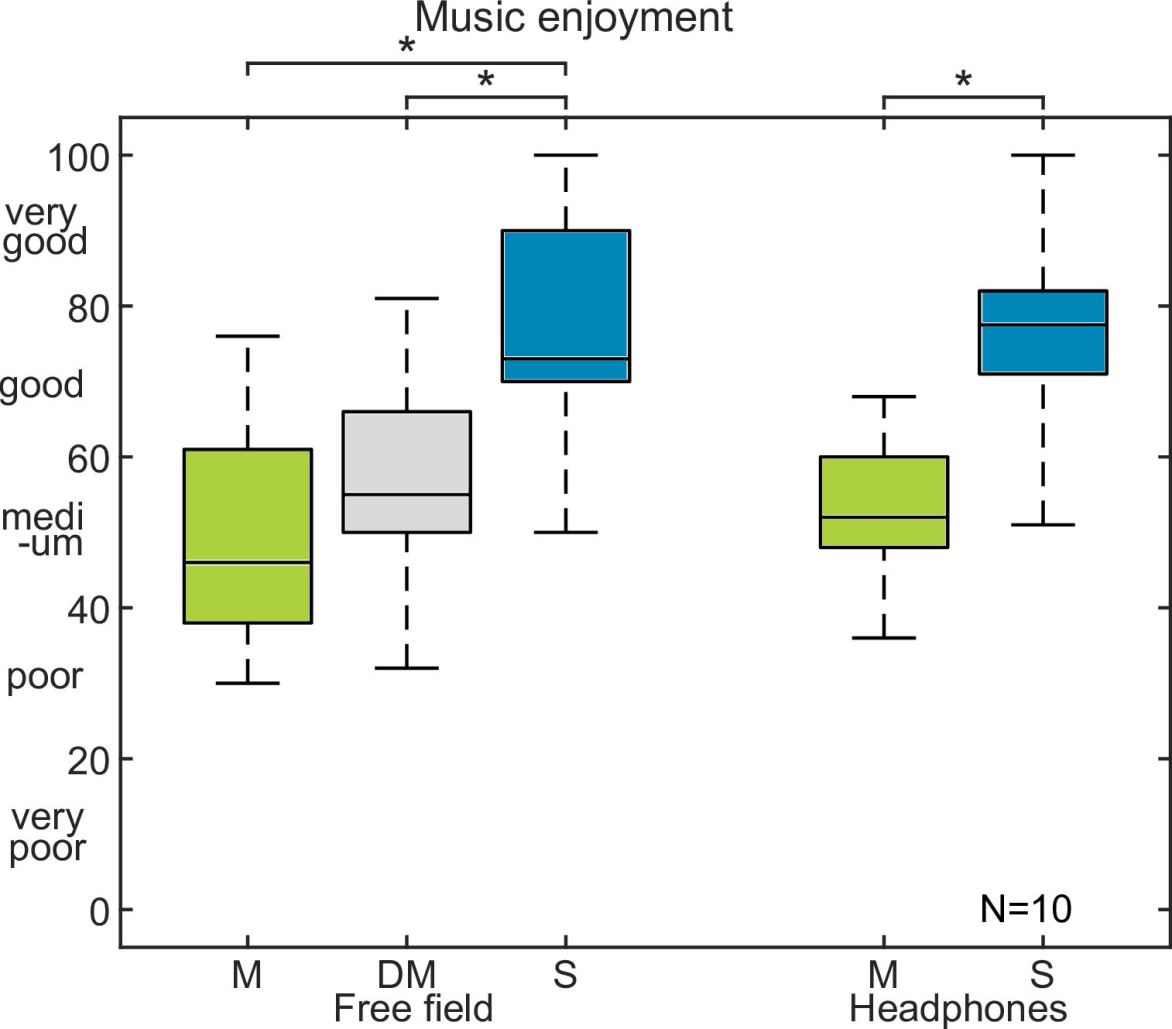
Results of experiment 2 with normal hearing listeners. Music enjoyment in normal hearing listeners for mono (M), double-mono (DM) and stereo (S) in free field and mono and stereo with headphones.

In Fig 3 the NH group showed median music enjoyment in free field of 46 for mono, 55 for double-mono and 73 for stereo. With headphone presentation, the medians increased from 52 to 78. Wilcoxon statistics with a Bonferroni-Holm correction revealed significant differences for stereo versus mono in free field (p<0.05) as well as with headphones (p<0.05) and double-mono versus stereo in free field (p<0.05).

**Fig 4:**
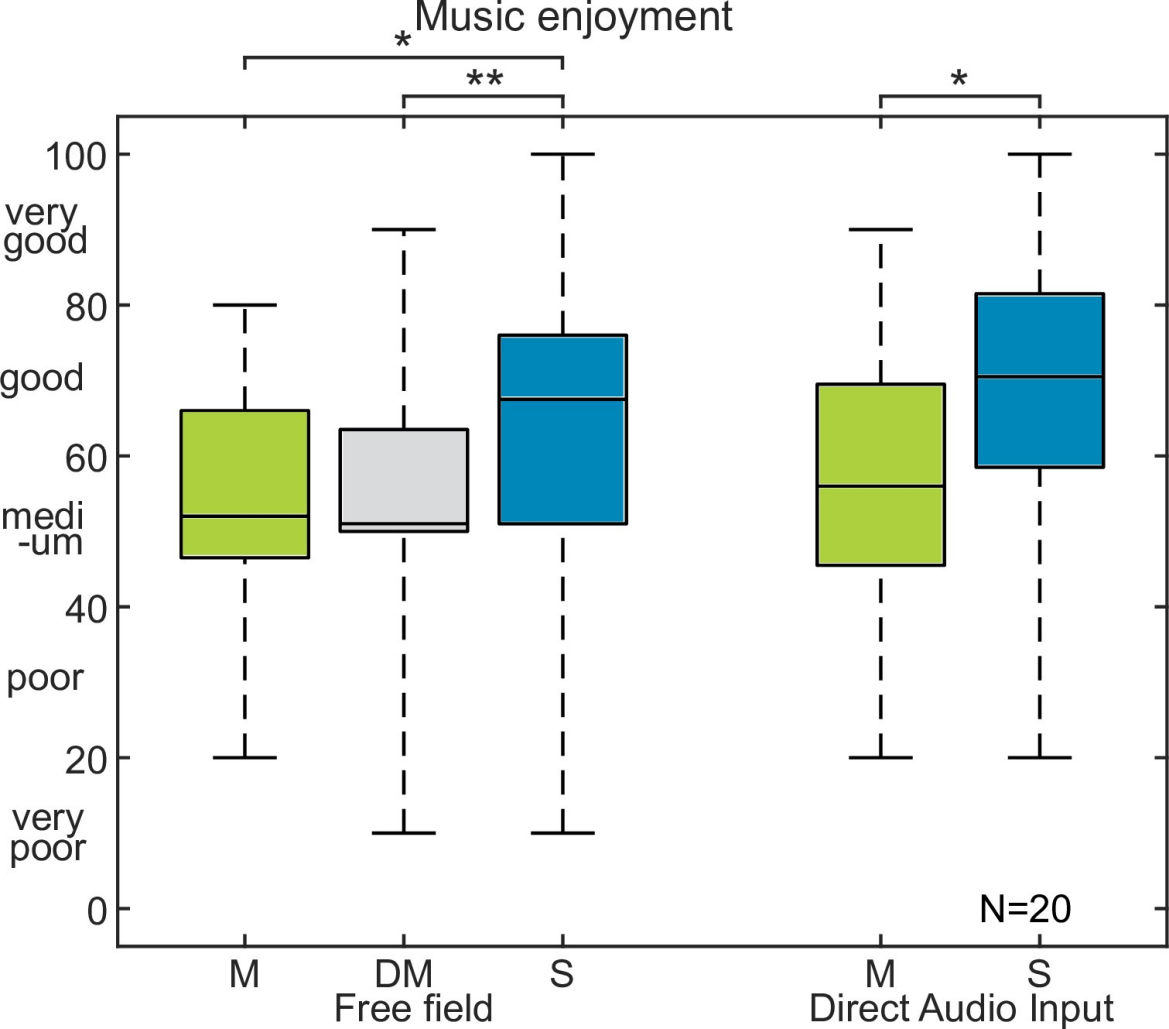
Results of experiment 2 with bilateral CI users. Music enjoyment in the bilateral CI group for mono (M), double-mono (DM) and stereo (S) in free field and mono and stereo with the direct audio input. Note that the data from both bilateral CI groups were pooled.

The music enjoyment for the pooled data from both bilateral CI groups is shown in Fig 4. Music enjoyment in free field was rated with 52, 51 and 68 for mono, double-mono, and stereo in free field as well as with 56 and 71 for mono and stereo with direct audio input, respectively. Wilcoxon statistics with a Bonferroni-Holm correction revealed significant increase in music enjoyment for stereo music compared to mono (p<0.05) and double-mono (p<0.01) in free field, as well as for stereo compared to mono with direct audio input (p<0.05).

**Fig 5:**
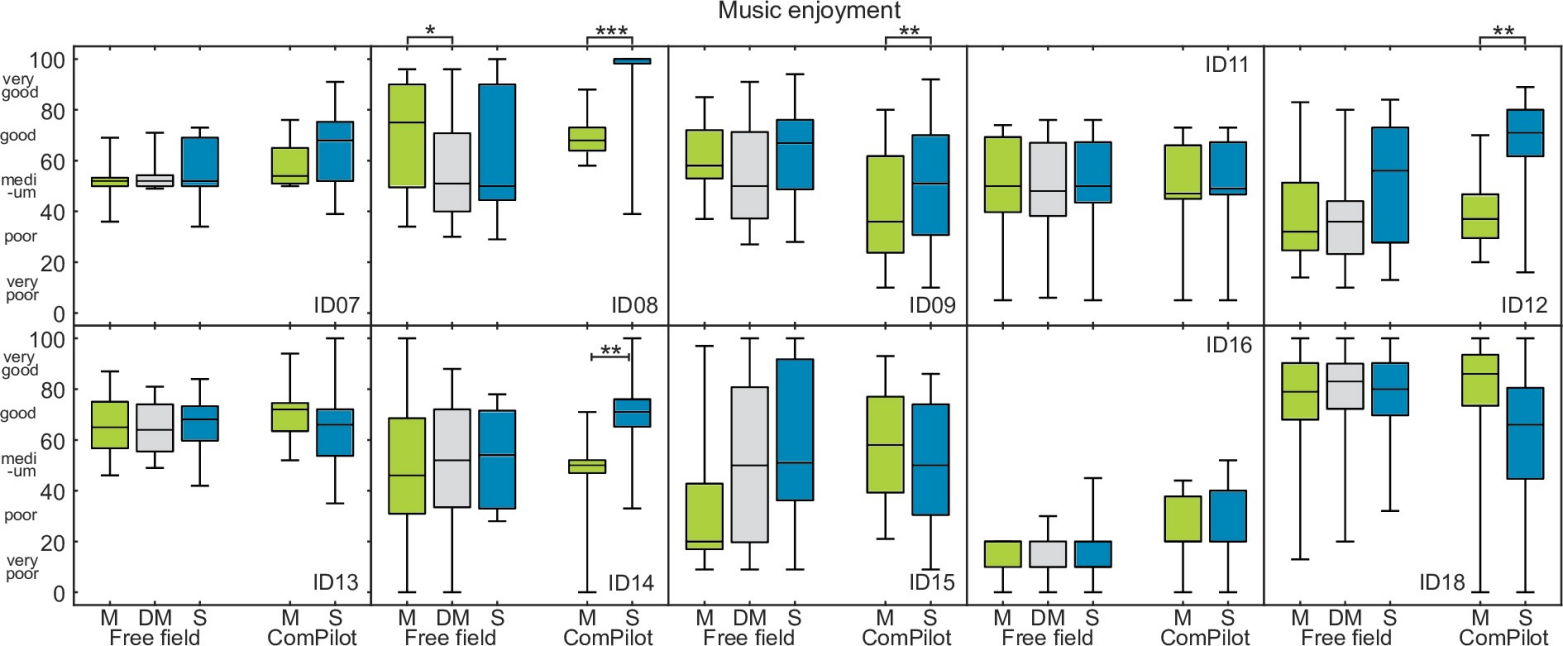
Results of experiment 2 with bilateral ComPilot group. Individual results for the ComPilot group of bilateral CI users. Each box consists of the responses of 25 music pieces. The boxes represent in free field mono (M), double-mono (DM) and stereo (S) as well as with ComPilot mono and stereo.

**Fig 6:**
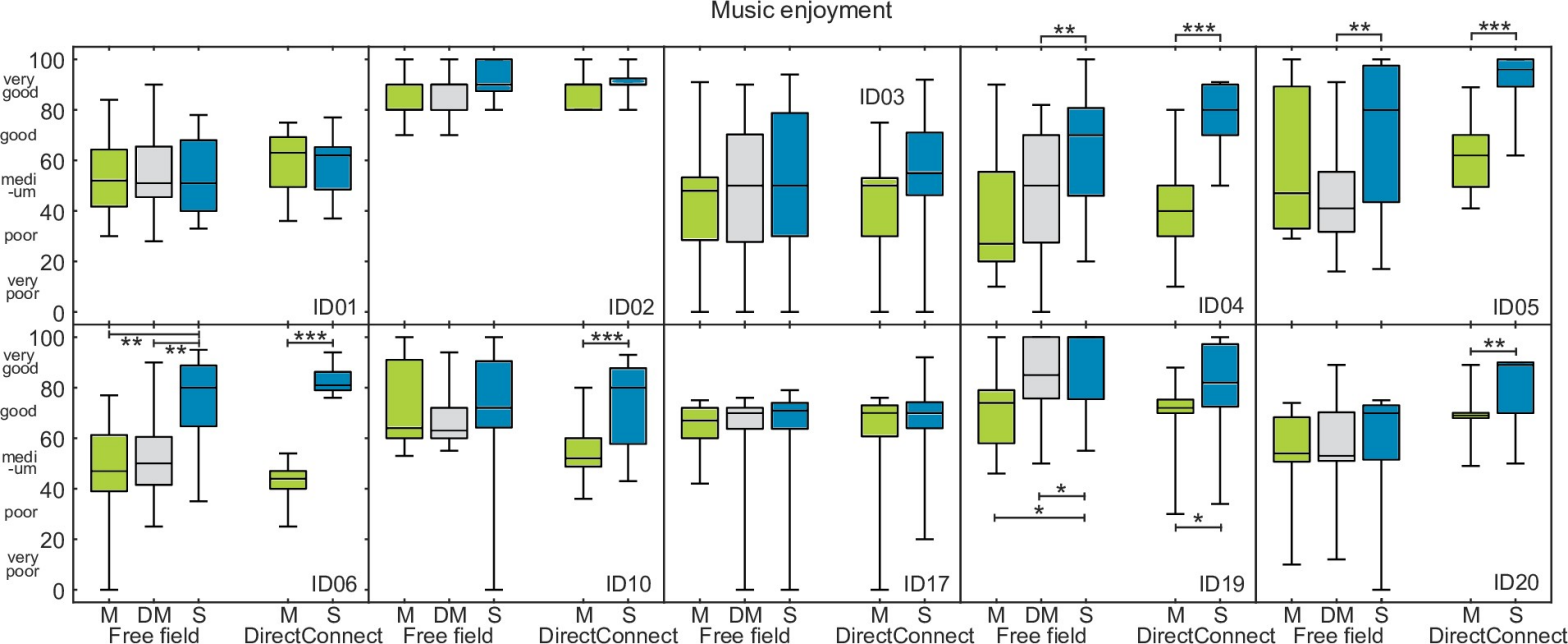
Results of experiment 2 with bilateral DirectConnect group. Individual results for the DirectConnect group of bilateral CI users. Each box consists of the responses of 25 music pieces. The boxes represent in free field mono (M), double-mono (DM) and stereo (S) as well as with DirectConnect mono and stereo.

The individual responses from Fig 4 were further analyzed for the CP and DC group shown in Figs 5 and 6, respectively.

For each user, the individual scale values for all 25 songs are shown as boxplots. Friedman tests were conducted for all subjects and a Bonferroni-Holm correction was applied. For each subject with a significant difference on the Friedman test, post-hoc Wilcoxon statistics were applied with Bonferroni-Holm correction to compare the individual conditions. Significant differences between conditions are indicated with asterisks.

In the CP group, 4 subjects had increased music enjoyment for stereo music with the CP, whereas none had increased music enjoyment for stereo in free field. For the DC group 6 subjects had increased music enjoyment for stereo with DC, whereas 3 of them also had increased music enjoyment for stereo music in free field.

**Fig 7:**
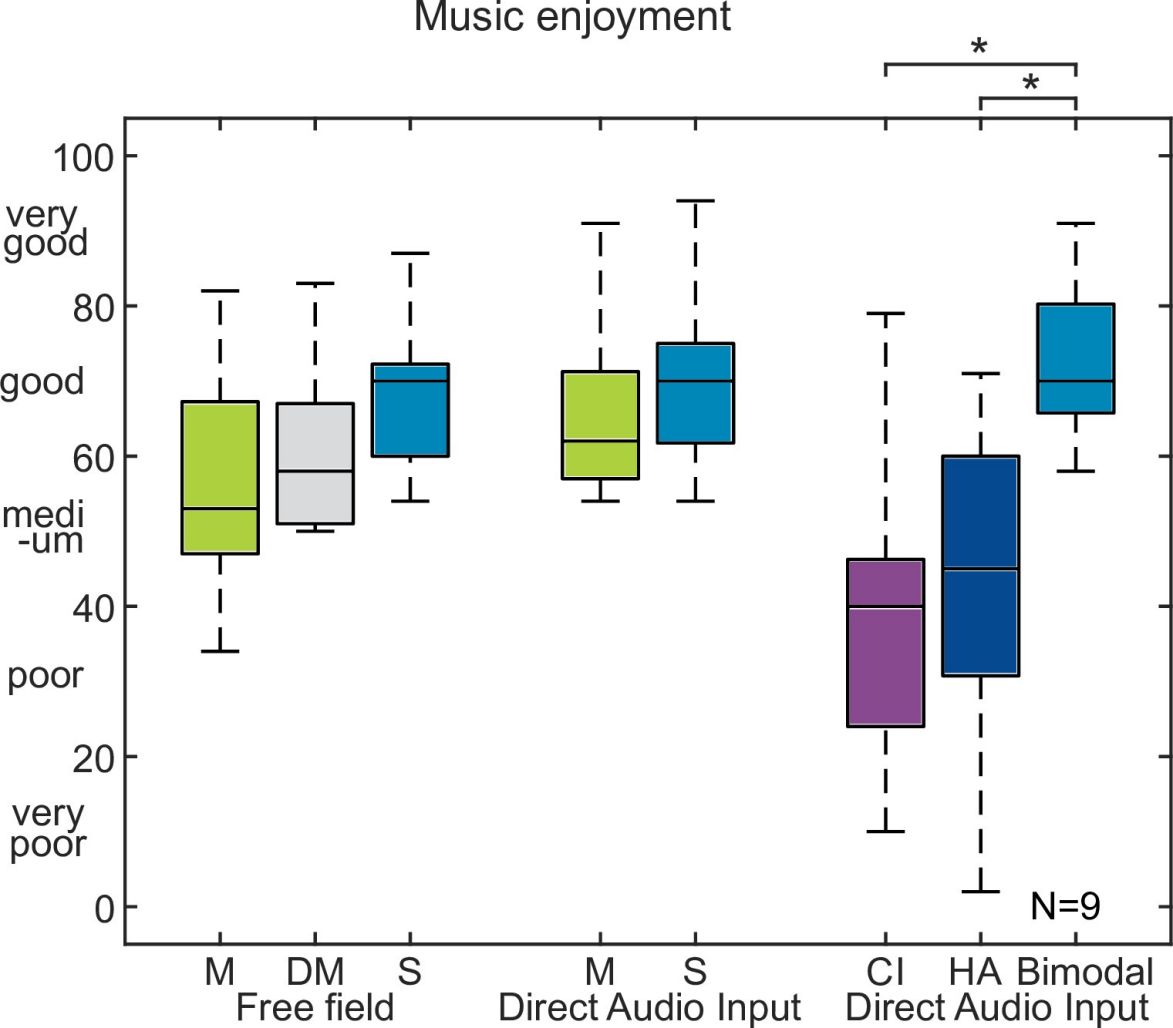
Results of experiment 2 and 3 with bimodal CI users. Music enjoyment in bimodal listeners for mono (M), double-mono (DM) and stereo (S) in free field and mono and stereo with direct audio input.

The results from Experiment 2 and 3 for the bimodal CI users are shown in Fig 7. Music enjoyment changed in free field from 53, 58 to 70 for mono, double mono and stereo, respectively. For direct audio, music enjoyment was 62 and 70. Music enjoyment for the CI alone, HI alone and the bimodal case increased from 40, 45 to 70. Significant differences for the bimodal group were found only for HA vs. bimodal (p<0.05) and CI vs. bimodal (p<0.05). As no difference was observed between CP and DC, the data are not shown here.

### Correlation between stereo detection and music enjoyment in bilateral CI users

In order to assess a possible dependence between stereo detection and music enjoyment, Pearson correlation coefficients were calculated for the bilateral CI user group.

In the first step, the number of wrongly identified stereo versions from Experiment 1 was counted for each of the 25 music pieces. For this purpose, the data from both bilateral CI groups were pooled for free field and direct audio input. Therefore, the stereo version of one music piece could be mistaken by maximal 20 if all bilateral CI users could not identify it.

In the next step, the difference between enjoyment scale values for stereo and mono versions within one listening condition (free field or direct audio input) was calculated for each music piece and for each bilateral CI user. Afterwards, the median was taken for each music piece pooled across all users for free field and the direct audio presentation.

**Fig 8:**
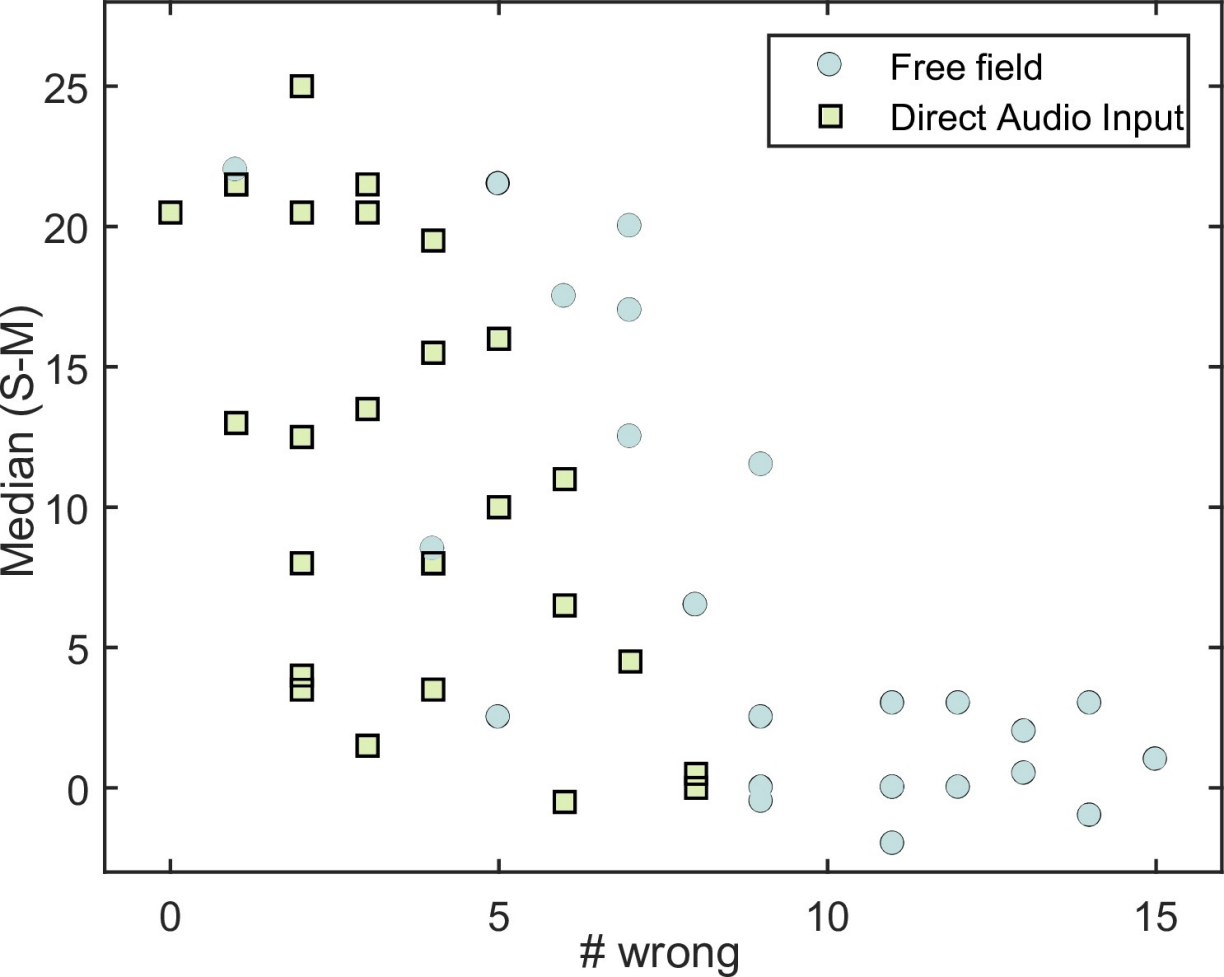
Comparison of stereo detection and music enjoyment. Median difference between stereo and mono plotted against the number of wrong detection for each music piece in free field (filled dots) and with direct audio input (open squares).

In the last step, the median music enjoyment was plotted against the number of wrong detections for each music piece in Fig 8, for free field (filled dots) and direct audio presentation (open squares). Each data point represents one music piece. The number of wrong detections is shown on the x-axis. For each song, the scale difference between stereo and mono versions is shown on the y-axis. For the free field condition, a significant negative Pearson correlation coefficient of -0.74 (p<0.001) was calculated. For direct audio presentation, a significant negative Pearson correlation coefficient of -0.56 (p<0.01) was calculated. For the free field condition, the less often subjects could detect a stereo song (i.e., > 10 number wrong), the lower was the median enjoyment scale difference between mono and stereo (i.e., the scale difference is close to 0).

The procedure described above was not applied to bimodal users, as no improvement in stereo detection (see Fig 1) and no difference between mono and stereo (see Fig 7) was obtained.

## Discussion

### Stereo detection

The results from Experiment 1 showed that CI users have some difficulties in perceiving the stereo effect in music listening, especially in free field conditions. NH listeners found this task easy, identifying 96% of the stereo pieces correctly, compared to a less than 52% identification rate for bilateral and 44% for the bimodal CI user group. This was despite the ideal lab conditions in which the experiment was conducted with low reverberation in the measurement room and the ideal position of the speakers for stereo listening. In contrast, stereo detection when using direct audio presentation was much closer to the normal group scores in the free field for the bilateral CI group (86%), although when using headphones the NH reached perfect detection in the test. There was a significant improvement for the bilateral group in median and individual (19 out of 20, not shown here) detection rates, when direct audio input was used. When the data was analyzed by song and the number of wrong answers from the bilateral groups pooled, the improvement in performance with direct audio input was present for all but one song (not shown here).

The enhanced detection rate for stereo music with direct audio presentation results from its ability to bypass all room acoustics and to minimize cross-talk from the speakers to the ears and microphones. Cross-talk means that sounds from the left loudspeaker are processed by both left and right ears and vice versa. Low frequencies from one loudspeaker are diffracted round the head to the contralateral ear and thus remain largely unaffected, whilst the high frequencies are enhanced at the near ear and reduced at the farther ear. With direct audio presentation, the left and right channels are presented directly to the left and right ears respectively omitting any cross-talk and resulting in enhanced ILDs and ITDs compared to the free field condition. Room reverberation was also a factor in the free field results as, even though it was low, studies of bilateral CI users show that their ability to use ITD and ILD cues to localize sound was considerably disrupted when even very low levels of reverberation were present [31]. There is no doubt that differences in CI user’s ability to detect ILD and ITD cues exist and some individuals are better able to detect and maintain discrimination of these cues in less than ideal conditions [21, 31, 32]. Thus, individual variations in the ability to detect stereo input could also be expected.

Some of the bimodal CI users showed benefits in stereo detection with direct audio presentation compared to free field presentation whereas others did not show any benefit. As the bimodal CI users were fitted with the bimodal fitting formula which avoided gain in dead regions [29], audibility on the HA side was ensured only for low frequencies, which were not delivered by the CI to the auditory nerve. As a result, low frequency parts of music may be perceived with the HA ear only whereas high frequency parts may be perceived with the CI ear only. This resulting little interaural frequency overlap might have resulted in poorer stereo detection in free field. With direct audio presentation bypassing the room acoustics stereo, detection improved only little compared to the bilateral CI users. However, due to the small sample size and different PTAs in the bimodal group, a strong conclusion on the role of residual hearing or aidable range for stereo detection in free field and direct audio presentation cannot be drawn in this study.

Furthermore, patient specific audiometric and anamnestic (demographic) data of bilateral as well as bimodal CI users was analyzed for any correlations with stereo detection. However, no correlations between the two domains were found.

### Music enjoyment

For NH listeners, there was a significant improvement in music enjoyment between stereo and mono for both listening conditions. In addition, the double-mono resulted in some non-significant increase in music enjoyment in the NH group, who reported that double-mono led to the impression that they were surrounded by music, whereas mono was perceived from front.

The pooled data from the bilateral CI users showed that bilateral CI users were able to perceive the stereo effect in free field as well as with direct audio input. However, further data analysis for both groups revealed large individual differences. Individual data from bilateral CI users from the CP group did not show any improvements for stereo in free field. In the DC group, there was some individual significant improvement in enjoyment for stereo compared to mono in the free field condition. The DC group had a higher stereo detection rate than the CP group. Correlation analysis confirmed that the easier stereo detection was, the higher was the enjoyment rating. Because the music enjoyment between mono and double-mono did not differ in both bilateral CI users group, it was assumed that they did not hear any difference when the signal was presented from two separated speakers in contrast to the NH listeners. Note, that although the scaling values for music enjoyment of NH listeners and CI users (Figs 3 and 4) appeared similar, it did not allow to conclude that music enjoyment between NH listeners and CI users was the same in this study. Rather these similar values were likely attributed to the nature of this listening experiment using a rating scale with a limited rating range. Given that the music pieces were unknown to all subjects, and that each music piece with mono and stereo versions should be compared with each other, the subjects were likely reluctant to use the endpoints of the complete scale and therefore showed a tendency to cluster around the center of the scale [33]. As a consequence, an absolute rating of music enjoyment between NH listeners and CI users could not be derived from the study, rather only the comparison of relative scale differences of stereo and mono versions within one group was possible. Any methods such as training or pilot experiments to the test material was not applicable for bias reduction as music enjoyment is a subjective perception and cannot be anchored as in traditional audio quality experiments [33].

Analysis of individual results showed that 10 out of the 20 bilateral subjects showed a significant improvement in enjoyment for stereo with the direct audio input, but only three had increased enjoyment in free field. One subject (ID18) rated stereo listening worse than mono with direct audio. The subject reported that he heard the stereo effect for the first time with the CP and found it disturbing to hear different signals on both ears. Since this subject only perceived stereo for the first time during the study, it may be that with further listening experience the signals would become more fused.

The data from both bilateral CI user groups indicate that stereo listening contributes to music enjoyment compared to mono listening with the direct audio input. However, it only benefited a few subjects in the free field condition. Stereo detection was improved with direct audio presentation resulting in increased music enjoyment.

For bimodal CI users no clear benefit of the stereo effect on music enjoyment could be observed. Similar to stereo detection, the small sample size as well as the different PTA hearing losses did not allow for strong conclusions. Nevertheless, Experiment 3, in which music enjoyment was assessed for CI alone, HI alone and both devices together, showed that music enjoyment increased from CI, HI to both devices. The increase is in line with [34], who reported the same order of increase in pleasantness and performance in bimodal CI users, which is likely attributed to improved pitch and melody perception via the HA [11].

Again, no correlation between patient specific audiometric, anamnestic (demographic) data and music enjoyment was found.

### Clinical relevance of the setup

Stereo listening in real life situations is probably worse than shown, as a loudspeaker setup with the optimal position of the speakers for stereo listening and a sound damped measurement room were used for this study. In real rooms, loudspeakers and the listening position are in most cases not optimal and reverberation and noise will reduce the perception of the stereo effect. To perceive the stereo effect when listening to music, direct audio is recommended for CI users, because direct audio bypasses all room acoustics, resulting in enhanced interaural level differences for CI users. Direct audio can be achieved with headphones using the T-Mic available in the Advanced Bionics system [35], the wired DC together with Harmony processors or the wireless CP with Naída CI.

## Conclusions

Bilateral CI users were able to detect the stereo effect and this contributed to overall music enjoyment with direct audio presentation. In free field, stereo perception remains difficult even in optimal listening conditions. Direct audio presentation resulted in improved stereo perception and increased music enjoyment.

Bimodal CI users were not able to detect the stereo sound and, therefore, the presentation of stereo signals did not increase music enjoyment compared to mono presentation. Nevertheless, music enjoyment was increased by listening to music with the HA and CI together compared to listening with the CI alone significantly.

## Supporting information

S1 Table Data from experiment 1, 2 and 3. (XLSX)
